# BART, the new robotic assistant: big data, artificial intelligence, robotics, and telemedicine integration for an ICU 4.0

**DOI:** 10.1186/s44158-024-00180-4

**Published:** 2024-07-12

**Authors:** Maria Grazia Bocci, Raffaella Barbaro, Valentina Bellini, Christian Napoli, Luigino Jalale Darhour, Elena Bignami

**Affiliations:** 1 National Institute for Infectious Diseases, Lazzaro Spallanzani, IRCCS, Via Portuense, 292, 00149 Rome, Italy; 2 Anesthesiology, Critical Care and Pain Medicine Division, Department of Medicine and Surgery, University of Parma, Viale Gramsci 14, 43126 Parma, Italy; 3 National Institute for Health, Migration and Poverty (NIHMP), Via Di San Gallicano 25, 00100 Rome, Italy; 4 Department of Medical Surgical Sciences and Translational Medicine, Sapienza University of Rome, 00189 Rome, Italy

**Keywords:** ICU, Big data, Artificial intelligence, Robotics, Telemedicine

## Abstract

We are in the era of Health 4.0 when novel technologies are providing tools capable of improving the quality and safety of the services provided. Our project involves the integration of different technologies (AI, big data, robotics, and telemedicine) to create a unique system for patients admitted to intensive care units suffering from infectious diseases capable of both increasing the personalization of care and ensuring a safer environment for caregivers.

New technologies are transforming the world of healthcare. Health 4.0 is a term that describes the use of Industry 4.0 technologies with the aim of improving the quality and security of healthcare services provided [1]. That means not only artificial intelligence (AI) but also the Internet of Things (IoT), big data, Cloud, etc. [2]. In the wake of the recent pandemic, there has been a great deal of interest in the potential use of these new tools in the context of infectious diseases. Some technologies, such as those related to telemedicine, have accelerated rapidly, becoming real clinical applications within a few months. Robotics provided another interesting insight. Within the existing subgroups, medical robots belong to the service robots. The term refers to a robot with a range of tasks designed to create a safer environment for caregivers and patients, decrease the physical demands on human workers, and ultimately provide a higher level of care. These attributes are well aligned with the context of infectious diseases, where issues about clinical management, workforce safety, and prevention of disease spread co-exist. There are several examples of robotic systems used effectively during the SARS-CoV-2 pandemic. At the end of 2020, the Mayo Clinic team proposed a collaborative robot capable of performing five simple tasks [3]. In 2021, Gao et al. published a very interesting review about the role of robotic technologies in combating infectious diseases [4]. Four main areas of interest have been identified: clinical care, public safety, laboratory, out-of-hospital care, work, and education continuity.

However, as described by Pang et al., one of the major attributes of the Health 4.0 novel approach is the change of the paradigm of systems design, shifting from one to multiple loops [1]. Too often, technologies are developed in parallel, disconnected from each other. Instead, what is needed is a synergy between computer science, engineering, and robotics that can perform intelligent, integrated, multi-loop tasks [5]. We are proposing a project capable of integrating different technologies (AI, big data, robotics, and telemedicine) to develop a unified tool for critically ill patients admitted to the intensive care unit (ICU) suffering from infectious diseases. The aim is twofold: to improve the quality and personalization of services and, at the same time, increase the safety of clinical staff. The study setting is the Lazzaro Spallanzani National Institute for Infectious Diseases (INMI), located in Rome, in collaboration with the National Institute for Health, Migration, and Poverty (NIHMP), a juridical body under the Italian Ministry of Health, which provides health care assistance, research, and training to offer a new model of aid to vulnerable population admitted to the ICU of INMI. As a matter of fact, in Italy, infectious diseases are still common, especially in poor and disadvantaged groups, in which the lack of a social and familiar network can worsen complications and rehabilitation. During hospitalization, it is still essential to guarantee an efficient system for maintaining and improving the cognitive functions of the patients, especially when they are more vulnerable. AI stimulates patients admitted to the ICU for the complications of infectious diseases to facilitate their discharge and social reintegration.

Three parts form the project’s anatomy: body, heart, and brain (Fig. 1).

**Fig. 1:**
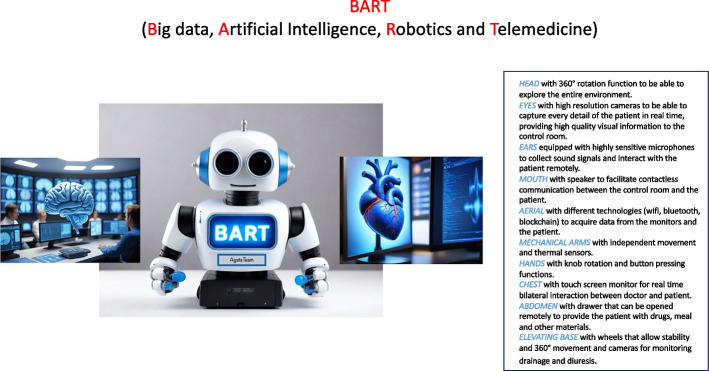
This figure represents the three *anatomical* parts of our project. The anthropomorphic robot is the body; each of its technical components will have specific functions, as described in the box. The platform represents the heart, which collects, analyzes, and processes data using artificial intelligence algorithms to guarantee tailor-made care for patients. The control room is the brain, where data collected by the body and processed by the heart flow converged. Here clinicians can analyze data and algorithm outputs, interact with patients, and give orders to the robots. (The images were created by *YouCam AI Pro* App)

## Body

An anthropomorphic robot represents the body. The robot will perform several basic functions, such as distributing pre-selected therapy and meal delivery. As already documented in the literature, simulations will be applied to identify the most critical nodes of interaction between patients and clinical staff that could most benefit from the robot’s use to limit infectious risks among healthcare workers [6].

The robot will also have two other abilities. It will be a nursing robot that will enter the patient’s room and, thanks to its cameras and transmission capabilities, collect the information available (data on the patient, on any equipment, etc.). In addition, it will be equipped with a remote system capable of creating interaction and communication with clinical staff [7]. A positive impact of robotic telepresence on reducing feelings of loneliness in awake patients will be expected [8].

## Heart

The heart is identified in a platform capable of collecting all available information. This data will come partly from the robot and partly from the IoT-equipped tools (monitors, ventilators, pumps for infusions, etc.). Like what happens with blood in the human body, all data will be channeled into a single structure that can collect data directly from the patient’s room and integrate it with the rest of the IT systems (laboratory tests, radiology notes, etc.). The raw data will then be processed so that it can be used to fuel advanced analytical systems, such as those based on machine learning. The ability of such algorithms to analyze highly complex data and to improve over time is one of the criteria for their adoption [9]. The algorithms will perform various tasks, from diagnosing and prognosticating to personalizing treatments.

## Brain

The control room can be considered as the brain of the whole system. In fact, the project foresees a control room where it will be possible to view, through user-friendly interfaces, the images transmitted by the cameras and data from the IoT systems. This network will be able to provide various interactions, from the simplest, such as monitoring infusions, to the most complex, such as giving instructions to the robot.

There are several challenges to overcome [10]. Unifying signals from different sources will be among the most important. Data integration and interoperability are essential for digital transformation [11]. Patient privacy will be another critical issue. Indeed, the platform will collect highly sensitive information, such as images. Careful consideration must, therefore, be given to ensure the highest security standards. Methods such as distributed technologies may be used to protect the integrity and quality of the network [12]. A further important step in the project will be solid clinical implementation planning. Involving the entire research team and all professionals, including nurses and medical and legal professionals, in a constant two-way dialogue is essential to creating the best conditions for an actual, successful, safe, real-life implementation [13].

## Data Availability

No datasets were generated or analysed during the current study.
